# Serum caspase-cleaved cytokeratin-18 fragment as a prognostic biomarker in hematological patients with febrile neutropenia

**DOI:** 10.1007/s10238-021-00734-8

**Published:** 2021-07-13

**Authors:** Carina Intke, Sini Korpelainen, Marika Lappalainen, Matti Vänskä, Sari Hämäläinen, Kari Pulkki, Esa Jantunen, Auni Juutilainen, Anna-Kaisa Purhonen

**Affiliations:** 1 Department of Medicine, Institute of Clinical Medicine/Internal Medicine, Kuopio University Hospital, P.O.B. 100, 70029 KYS Kuopio, Finland; 2 Siun Sote – Hospital District of North Carelia, Joensuu, Finland; 3 Department of Internal Medicine, Tampere University Hospital, Tampere, Finland; 4 Department of Internal Medicine, Seinäjoki Central Hospital, Seinäjoki, Finland; 5 Diagnostic Center, Helsinki University Hospital, Helsinki, Finland; 6 Clinical Chemistry and Hematology, University of Helsinki, Helsinki, Finland; 7 Institute of Clinical Medicine/Internal Medicine, University of Eastern Finland, Kuopio, Finland; 8 Department of Internal Medicine, South Carelia Central Hospital, South Carelia Social and Health Care District (Eksote), Lappeenranta, Finland

**Keywords:** Caspase-cleaved cytokeratin-18, Febrile neutropenia, Severe sepsis, Acute myeloid leukemia, Non-Hodgkin lymphoma, Procalcitonin

## Abstract

The study aim was to determine the benefit of the measurement of serum caspase-cleaved cytokeratin-18 (CK-18) fragment as a prognostic marker of febrile neutropenia (FN) in hematological patients. The study population consisted of 86 consecutive patients with FN who received intensive chemotherapy for hematological malignancy at the adult hematology ward of Kuopio University Hospital. Twenty-three patients (27%) had acute myeloid leukemia, and 63 patients (73%) were autologous stem cell transplant recipients. Serum caspase-cleaved CK-18 fragment M30, C-reactive protein (CRP) and procalcitonin (PCT) were measured at the onset of FN (d0), on day 1 (d1), and on day 2 (d2). Eight patients (9%) developed severe sepsis, including three patients with septic shock. Eighteen patients (21%) had a blood culture-positive infection. Serum CK-18 fragment peaked on the first day after fever onset in patients with severe sepsis. Higher CK-18 level was associated with severe sepsis, intensive care unit treatment, and fatal outcome, but not with blood culture positivity. In ROC curve analysis, d1 serum CK-18 fragment predicted severe sepsis with an area under the curve (AUC) of 0.767, CRP with an AUC of 0.764, and PCT with an AUC of 0.731. On d2, the best predictive capacity was observed for CRP with an AUC of 0.832. The optimal cutoff of caspase-cleaved CK-18 fragment M30 for predicting severe sepsis was 205 U/L on d1. In hematological patients, serum CK-18 fragment was found to be a potential prognostic marker of severe sepsis at early stages of FN.

## Introduction

Febrile neutropenia (FN) is a common complication in hematological patients receiving intensive chemotherapy for acute myeloid leukemia (AML) or autologous stem cell transplantation (ASCT). Outcome in patients with neutropenic sepsis may be fatal, so the diagnosis must be prompt and antimicrobial treatment adequate and timely.

Cytokeratins (CK) are structural proteins found in epithelial and parenchymal cells. They form the cytoplasmic network of intermediate filaments playing a role in apoptosis, mitosis, cell cycle progression, and cell signaling [1, 2]. CK-18 has been investigated in several acute and chronic disease states [3–5], as well as in hepatology and histopathology [6–11]. In cancer research, it has been explored as a prognostic apoptosis-related marker [12]. CK-18 and its fragments are expected to correlate with tumor mass, tumor stage, and response to chemotherapy, as demonstrated in breast, colorectal, gastric, endometrial, lung, and prostate cancers among others [12–15].

Apoptosis is a form of programmed cell death, triggered by cysteine proteases of the caspase family. In apoptosis the dying cells are phagocytized before undergoing membrane damage, in contrast to other forms of programmed cell death [16]. Apoptosis is considered a vital component of various processes, including normal cell turnover, proper development and function of the immune system, hormone-dependent atrophy, embryonic development, and chemical-induced cell death [17]. Apoptosis is also a potential mechanism in sepsis and its complications [18, 19]. Several biomarkers for apoptosis have been recognized [20]. In apoptosis, intracellular CK-18 is cleaved by caspase-3 at two sites and the released fragments can be measured from blood using antibody against exposed M30 neoepitope [1]. Later in the results section, we refer with “CK-18” to caspase-cleaved CK-18 fragment quantified by M30 epitope concentration.

Levels of CK-18 were reported to be higher in patients with sepsis than in trauma patients and healthy controls [21, 22]. In critically ill patients with and without sepsis, the levels of CK-18 correlated with biomarkers of renal injury, liver injury, and high mortality [21, 22]. In patients with sepsis, high CK-18 levels have been observed to associate with mortality [23]. Also high caspase-3 levels have been found to be linked with early mortality of patients with severe sepsis [24].

CK-18 might be an especially interesting biomarker for hematological patients with FN as the intensive chemotherapy results both in cell death and mucosal injury. Furthermore, CK-18 is associated with organ failure in septic patients, an unfortunate consequence of FN. There are no previous studies on the use of caspase-cleaved CK-18 fragment levels as a prognostic marker in FN of hematological patients. The aim of this study is to evaluate the use of serum CK-18 fragment level as an early marker of severe complications of FN and to compare its performance with that of the conventionally used biomarkers C-reactive protein (CRP) and procalcitonin (PCT).

## Patients and methods

### Patients

The inclusion criteria were fulfilled if a patient had FN after intensive chemotherapy for AML or if the patient was an ASCT recipient. The study population consisted of 86 consecutive patients with FN who received intensive chemotherapy for hematological malignancy between December 2009 and November 2012 at the adult hematology ward of Kuopio University Hospital. Twenty‐three (27%) patients had acute myeloid leukemia and 63 (73%) patients were autologous stem cell transplant recipients (Table 1). None of the study participants had a diagnosed liver disease at the onset of the study. There were 55 males and 31 females with a median age of 61 years (18–70 years).

**Table 1:** Baseline characteristics during febrile neutropenia in 86 hematological patients after intensive chemotherapy

Sex	
Male	55 (64%)
Female	31 (36%)
Age	
Median (min, max), years	61 (18–70)
> 60 years	46 (53%)
Acute myeloid leukemia	23 (27%)
Autologous stem cell transplantation	63 (73%)
Non-Hodgkin lymphoma	43 (50%)
Multiple myeloma	17 (20%)
Hodgkin lymphoma	3 (3.5%)
Chemotherapy regimen	
BEAM	42 (49%)
HD-MEL	18 (20%)
IdAraC-Ida	9 (10%)
IAT	7 (8.1%)
MEA	4 (4.7%)
Carmustine-Thiotepa	3 (3.5%)
Mito-HDAraC	2 (2.3%)
HDAraC-Ida	1 (1.2%)
Positive blood culture	18 (21%)
Gram-positive	14 (16.3%)
Gram-negative	3 (3.5%)
Fungal sepsis	1 (1.2%)
Severe sepsis	8 (9.3%)
Septic shock	3 (3.5%)
Intensive care unit admission	6 (7.0%)
Death	3 (3.5%)

*BEAM*, carmustine, etoposide, cytarabine, and melphalan; *HD-MEL*, high-dose melphalan; *IAT*, idarubicin, cytarabine, and thioguanine; *IdAraC-Ida*, intermediate-dose cytarabine and idarubicin; *HDAraC-Ida*, high-dose cytarabine and idarubicin; *Mito-HDAraC*, mitoxantrone and high-dose cytarabin; *MEA*, mitoxantrone, etoposide, and cytarabine

### Clinical follow-up

All patients were carefully followed up at the hematology ward until recovery of neutropenia. Blood cultures (2–3 sets including 2 bottles/set) were drawn immediately at the beginning of FN. An additional sampling was done if fever persisted for more than three to five days. Blood pressure, oxygen saturation, respiratory frequency, heart rate, body temperature, urine output, fluid intake, as well as clinical signs of infection were followed up daily. Broad spectrum antibiotics were started after the samples for blood cultures were taken from cubital veins. The clinical data was recorded on a structured data collection form and included the hour and date of the start of fever, possible sites of infections, and hemodynamic parameters suggesting septic complications.

### Laboratory analyses

Blood samples for CK-18 fragments, CRP and PCT were collected at the onset of FN (d0) and on the following next two mornings (d1 and d2). Serum was separated and samples stored frozen at—70 °C until analysis. Blood cultures were processed using the automated blood culture system Bactec 9240 (Becton Dickinson, Sparks, MD, USA). The incubation time was 7 days for both aerobic and anaerobic bottles and 42 days for MYCO F/Lytic bottles. All samples were analyzed at the Eastern Finland Laboratory Centre in random order and blinded with respect to clinical data (except for real-time measurement of CRP).

Measurement of caspase-cleaved CK-18 concentration was performed with M30 Apoptosense® CK18 Kit (VLVBio, Nacka, Sweden). As declared by the manufacturer, the range of concentration of CK-18 was 40 – 1000 U/L, sensitivity 20 U/L, within-assay variation ≤ 10%, between-assay variation ≤ 10%, and total variation ≤ 10% for samples with concentrations over 200 U/L. . The cutoff given by the manufacturer was < 200 U/L in normal subjects and the 95th percentile was 251 U/L. According to the measurements in our laboratory, the intra-assay coefficient of variation (CV) was 7.7% at 164 U/L and the inter-assay CVs were 4.0% at 128 U/L and 3.3% at 634 U/L, respectively.

The concentration of CRP was measured with a Konelab60i Clinical Chemistry Analyzer (Lab systems CLD, Konelab, Helsinki, Finland) or Cobas 6000-analyzer (Hitachi, Tokyo, Japan). The between-run variations ranged from 2.3 to 4.3%. The upper reference limit of serum or plasma CRP of a healthy reference population is 5 mg/L.

Plasma PCT was analyzed with Cobas 6000-analyzer (Hitachi, Tokyo, Japan). The sensitivity of the assay was 0.06 µg/L*.* The within- and between-assay CVs for PCT analyses were 1.4 and 3.0% for 0.46 µg/L of PCT and 1.1 and 2.6% for 9.4 µg/L of PCT, respectively. The lower limit for PCT indicating a possible systemic infection is 0.5 µg/L.

### Definitions

Febrile neutropenia was defined using the criteria of the Infectious Diseases Society of America [25]. Neutropenia was considered as a condition of neutrophil count < 0.5 × 10^9^/L or with a predicted decrease to < 0.5 × 10^9^/L during the next 48 h. Fever was defined as a single oral temperature of 38.3 °C or a temperature of 38.0 °C sustained over a 1-h period. Sepsis was defined as a syndrome, in which systemic inflammatory response was present with infection [26, 27]. Severe sepsis was defined as sepsis with organ dysfunction according to the definition enforced during the study, and septic shock was considered to be present if there was hypotension (systolic arterial pressure < 90 mmHg, mean arterial pressure < 60 mmHg, or a reduction in systolic blood pressure of > 40 mmHg from baseline) despite adequate volume resuscitation in the absence of other causes of hypotension [28]. In February 2016, new definitions for sepsis and septic shock were announced [29], but in this prospective study design, we used the definitions that were available at the time of the study entry.

A single positive blood culture was considered significant if the microbe was a clinically relevant cause of infection. Common skin contaminants (e.g., coagulase-negative staphylococci) were considered significant only if they were found in two consecutive blood cultures or if there was a concurrent skin or catheter infection.

### Statistical analysis

Data analyses were conducted with IBM SPSS Statistics 27 for Windows (IBM, Armonk, NY, USA). Categorical variables were given as absolute counts or frequencies. Because of the log-normal distribution of PCT, logarithmic transformation was used for other than nonparametric analyses of PCT. Correlations between continuous variables were analyzed by the Spearman’s correlation test. CK-18, PCT and CRP were reported as medians with ranges and means with standard errors of mean according to the presence of complications of FN. The nonparametric Mann–Whitney U-test used to evaluate the differences of continuous variables between two groups and Kruskal–Wallis test between more than two groups. Receiver operating characteristics curve (ROC) analysis was carried out to evaluate the prognostic value of CK-18, PCT and CRP, providing values for the area under the curve (AUC) with 95% confidence intervals (CI). The significance of differences between AUC values was tested by the method described by Hanley and McNeil [30]. Youden’s indices (sensitivity + specificity—1) were calculated. Also weighted Youden’s indices were used for determining alternative cutoffs [31]. The weighted Youden’s index (J_w_) was defined as follows:

$$ J_{w}  = {\text{ }}2{\text{ }} \times {\text{ }}(\lambda  \times {\text{ sensitivity }} + {\text{ }}(1 - \lambda ){\text{ }} \times {\text{ specificity}}){\text{ }} - {\text{ }}1;{\text{ }}(0{\text{ }} \le \lambda  \le {\text{ }}1)$$

By definition, as the sum of the weights (λ and 1-λ) is 1, J_w_ ranges from -1 to + 1 (as also does unweighted Youden’s index), and is equal to unweighted Youden’s index when sensitivity and specificity have equal weights. The values λ = 2/3 and λ = 1/3 were used to emphasize sensitivity and specificity, respectively. A P value of less than 0.05 was considered significant.

## Ethical approval

Informed consent was obtained from all individual participants included in the study. This study was conducted according to the principles expressed in the Declaration of Helsinki and approved by the Ethical Board of North Savo Hospital District (100/2006).

## Results

Eight patients developed severe sepsis (9.3%) and six patients (7.0%) were treated at intensive care unit (ICU). Three patients developed septic shock and two of them died (Table 1). One patient without septic shock died during induction therapy for AML. Six out of eight patients with severe sepsis had positive blood culture findings. Altogether 18/86 patients (21%) had a positive blood culture finding. Three patients had Gram-negative bacteremia, fourteen patients had Gram-positive bacteremia, and one patient had candidemia. Blood culture positivity was not associated with plasma CK-18 level on d0-d2, but CRP level on d1-d2 and PCT level on d1 was significantly higher in blood culture positive than in blood culture negative patients (data not shown).

**Fig. 1:**
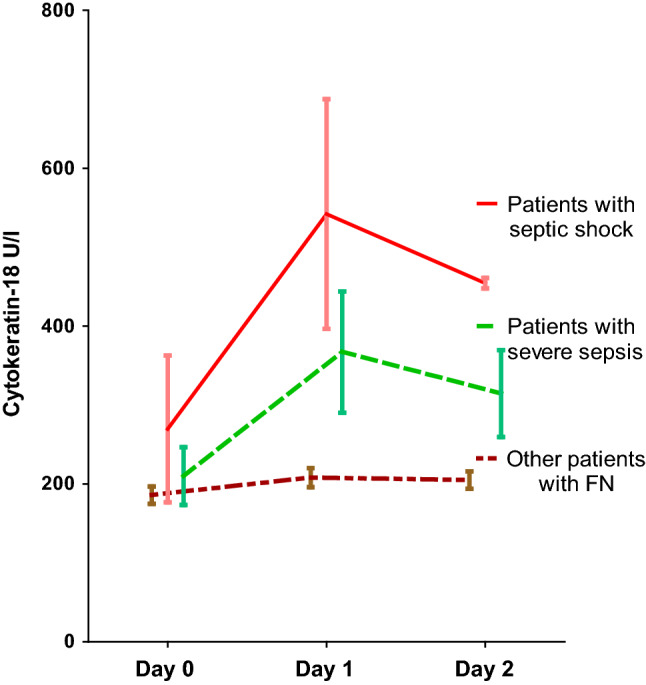
Levels of cytokeratin-18 (M30) (mean with standard error of mean) from day 0 to day 2 according to septic complications in 86 patients with febrile neutropenia

In patients with severe sepsis, the levels of CK-18 and PCT on d1, and the level of CRP both on d1 and d2, were higher than in patients without severe sepsis (Table 2). The level of CK-18 peaked on d1-d2 in patients with severe sepsis (Fig. 1, Table 2). On d0 the levels of CK-18, CRP and PCT were similar in patients with and without severe sepsis.

**Table 2:** Means and medians of cytokeratin-18, C-reactive protein and procalcitonin on day 0–2 from the onset of febrile neutropenia in 86 hematological patients according to the presence of severe sepsis after intensive chemotherapy

	No severe sepsis (*N* = 78)		Severe sepsis (*N* = 8)		*P* value^a^
	Mean ± SEM	Median (min, max)	Mean (SEM)	Median (min, max)	
*Cytokeratin-18 U/L*					
Day 0	186 ± 11	168 (46, 669)	210 ± 37	180 (113, 456)	0.326
Day 1	208 ± 12	179 (75, 566)	367 ± 77	309 (109, 697)	0.014*
Day 2	205 ± 11	188 (56, 722)	315 ± 55	337 (128, 461)	0.067
*C-reactive protein mg/L*					
Day 0	52 ± 6	36 (5, 286)	71 ± 22	64 (5, 212)	0.246
Day 1	88 ± 9	70 (9, 357)	162 ± 33	143 (48, 327)	0.015*
Day 2	114 ± 10	87 (7, 342)	245 ± 39	231 (129, 367)	0.004**
*Procalcitonin µg/L*					
Day 0	0.197 ± 0.026	0.128 (0.037, 1.74)	3.793 ± 3.547	0.214 (0.058, 28.62)	0.104
Day 1	0.856 ± 0.406	0.180 (0.029, 28.87)	6.041 ± 4.997	0.981 (0.075, 40.92)	0.032*
Day 2	0.776 ± 0.296	0.190 (0.036, 21.11)	5.655 ± 3.657	1.440 (0.073, 26.90)	0.057

^*^P < 0.05; **P < 0.01; ***P < 0.001; ^a^Nonparametric Mann–Whitney U-test

In patients who developed septic shock, the level of CK-18 was higher on d1-d2 than in patients who did not develop septic shock (Fig. 1), with median (range) values of CK-18 185 (169–456) U/L on d0, 677 (251–697) U/L on d1, and 454 (448–461) U/L on d2. The maximum d0-d2 levels of CK-18, CRP and PCT differed between the patients with and without septic shock (*P* values 0.001, 0.008 and 0.003, respectively). CK-18, CRP and PCT also differed between patients with and without a need for ICU treatment (*P* values 0.024, 0.005 and 0.012, respectively). Moreover, CK-18 and CRP differed between patients with and without fatal outcome during the hospital stay (*P* values 0.015 and 0.024, respectively), but the level of PCT was similar in these patient groups.

The biomarker levels were also evaluated according to the background malignancy. Higher serum levels of CK-18 were observed in patients with NHL than in patients with AML (Table 3). In contrast, the levels of CRP and PCT were similar in these two patient groups. Furthermore, the correlation of CK-18 with alanine aminotransferase, alkaline phosphatase, bilirubin, and body mass index was evaluated. No significant correlations were observed.

**Table 3:** Means and medians of cytokeratin-18, C-reactive protein and procalcitonin on day 0–2 from the onset of febrile neutropenia in 66 patients according to the diagnosis acute myeloid leukemia (AML) *vs.* non-Hodgkin lymphoma (NHL)

	Patients with AML (*N* = 23)		Patients with NHL (*N* = 43)		*P* value*
	Mean ± SEM	Median (min, max)	Mean (SEM)	Median (min, max)	
*Cytokeratin-18 U/L*					
Day 0	179 ± 29	132 (46, 669)	203 ± 13	181 (98, 456)	0.015*
Day 1	212 ± 35	141 (75, 677)	245 ± 19	203 (112, 697)	0.008**
Day 2	187 ± 29	187 (56, 722)	243 ± 16	210 (129, 492)	0.003**
*C-reactive protein mg/L*					
Day 0	78 ± 17	52 (6 286)	50 ± 6	39 (5, 151)	0.562
Day 1	118 ± 20	89 (14, 347)	109 ± 12	90 (25, 357)	0.908
Day 2	129 ± 16	121 (7, 273)	157 ± 16	151 (20, 367)	0.398
*Procalcitonin µg/L*					
Day 0	1.511 ± 1.235	0.137 (0.054, 28.62)	0.201 ± 0.022	0.157 (0.038, 0.678)	0.800
Day 1	4.000 ± 2.129	0.254 (0.075, 40.92)	0.444 ± 0.094	0.212 (0.039, 3.270)	0.607
Day 2	1.693 ± 0.986	0.234 (0.063, 21.11)	1.396 ± 0.660	0.277 (0.036, 26.90)	0.927

^*^P < 0.05; **P < 0.01; ***P < 0.001

**Fig. 2:**
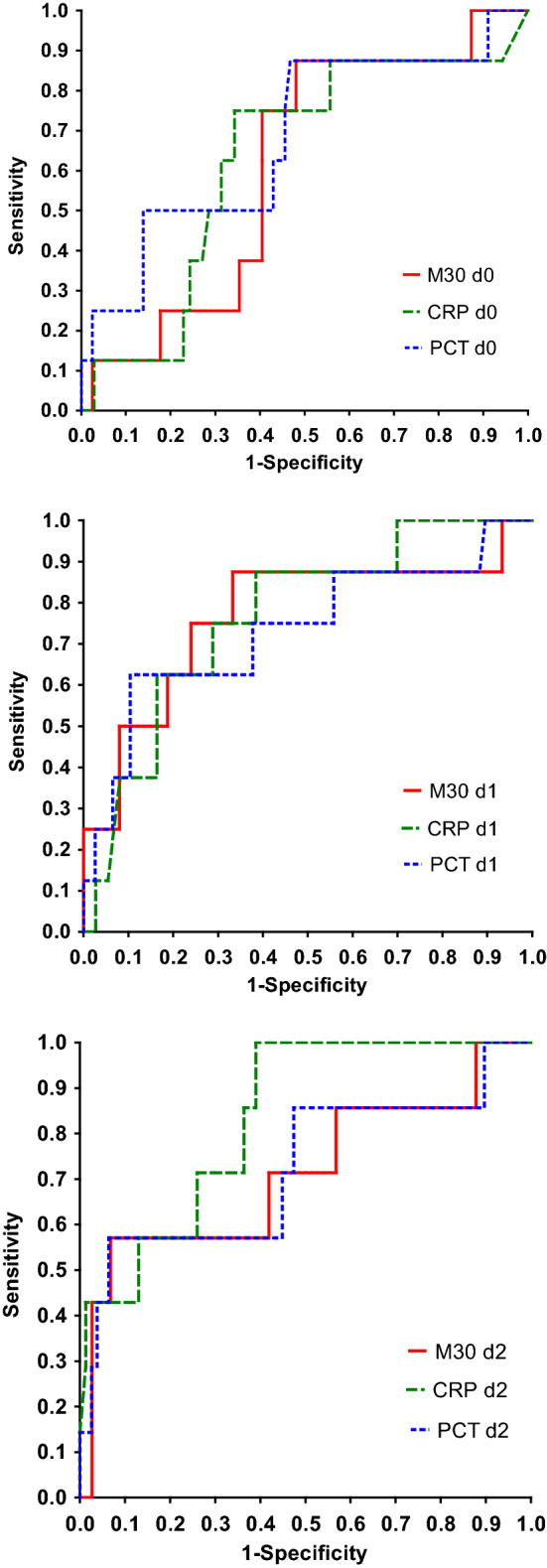
ROC curve analysis of cytokeratin-18 (M30). C-reactive protein (CRP) and procalcitonin (PCT) measured on day 0 (top), day 1 (middle), or day 2 (bottom) from the start of febrile neutropenia as a predictor of severe sepsis in 86 hematological patients after intensive chemotherapy

The results of the ROC curve analyses evaluating CK-18, CRP and PCT as predictors of severe sepsis are reported in Table 4 and Fig. 2. The AUCs on d1 after fever onset were highest for CK-18 (0.767, *P* = 0.011) and CRP (0.764, *P* = 0.001). On d2, only CRP showed a statistically significant AUC. Within maximum d0-d2 values, CRP showed the best performance with AUC 0.841 (*P* < 0.001), while the AUCs for CK-18 and PCT were 0.766 (*P* = 0.011) and 0.743 (*P* = 0.032), respectively. The differences between the AUCs were not statistically significant.

**Table 4:** ROC curve analysis of cytokeratin-18, C-reactive protein and procalcitonin on day 0–2 from the start of febrile neutropenia in predicting severe sepsis in 86 hematological patients after intensive chemotherapy

	Area under the curve	95% confidence interval	*P* value
*Cytokeratin-18*			
Day 0	0.606	0.428 – 0.783	0.243
Day 1	0.767	0.562 – 0.972	0.011*
Day 2	0.710	0.471 – 0.950	0.086
Max Day 0–2	0.766	0.562 – 0.970	0.011*
*C-reactive protein*			
Day 0	0.626	0.427 – 0.825	0.215
Day 1	0.764	0.604 – 0.924	0.001**
Day 2	0.832	0.701 – 0.963	< 0.001***
Max Day 0 – 2	0.841	0.723 – 0.958	< 0.001***
*Procalcitonin*			
Day 0	0.675	0.466 – 0.885	0.101
Day 1	0.731	0.516 – 0.946	0.035*
Day 2	0.718	0.477 – 0.959	0.077
Max Day 0 – 2	0.743	0.521 – 0.964	0.032*

^*^P < 0.05; **P < 0.01; ***P < 0.001

The diagnostic performance of CK-18, CRP and PCT from d0-d2 in FN is compared in Table 5. As sensitivity might be of special importance in search for cutoffs in this patient group at high risk for septic complications, also weighted Youden’s indices are reported. At the same level of sensitivity of 0.88 on d1, CK-18 showed the highest Youden’s index of 0.54, in comparison to those of CRP (0.49) and PCT (0.31). When the cut-offs based on optimized unweighted Youden’s index were used, PCT showed higher specificity but a lower sensitivity than CK-18 or CRP on d1. Weighting sensitivity had a disadvantageous effect (reduction of diagnostic odds ratio and Youden’s index) on the performance of CK-18 on d2 and PCT on d1 and d2. Of note, on day 1 the diagnostic odds ratio was above the level of ten for CK-18 both by sensitivity and specificity weighted cutoffs, but only by sensitivity weighted cutoff for CRP and specificity weighted cutoff for PCT.

**Table 5:** Diagnostic performance of serum cytokeratin-18 (CK-18), C-reactive protein (CRP) and procalcitonin (PCT) in predicting the development of severe sepsis after intensive chemotherapy on day 0, day 1, and day 2 after the onset of febrile neutropenia in 86 hematological patients. The alternative cut-offs are based on weighted Youden’s indices (J_w_) (more sensitive: λ = 2/3; less sensitive: λ = 1/3)

	CK-18		CRP		PCT	
	λ = 2/3	λ = 1/3	λ = 2/3	λ = 1/3	λ = 2/3	λ = 1/3
*Day 0*						
*Cut-off*	169 U/L*	*448 U/L*	51 mg/L*	*182 mg/L*	0.138 µg/L*	*0.273 µg/L*
Sensitivity	0.88	0.13	0.75	0.13	0.88	0.50
Specificity	0.51	0.97	0.65	0.97	0.53	0.86
LR +	1.80	4.88	2.16	4.31	1.84	3.55
LR-	0.24	0.90	0.38	0.90	0.24	0.58
DOR	7.37	5.43	5.63	4.79	7.76	6.09
Youden	0.39	0.10	0.40	0.10	0.40	0.36
J_w_	0.51	0.38	0.44	0.38	0.52	0.48
*Day 1*						
*Cut-off*	205 U/L*	*347 U/L*	86 mg/L*	*133 mg/L*	*0.154 µg/L*	0.887 µg/L*
Sensitivity	0.88	0.50	0.88	0.63	0.88	0.63
Specificity	0.66	0.92	0.61	0.83	0.43	0.89
LR +	2.59	6.17	2.25	3.75	1.55	5.94
LR-	0.19	0.54	0.20	0.45	0.29	0.42
DOR	13.7	11.3	11.0	8.33	5.37	14.2
Youden	0.54	0.42	0.49	0.46	0.31	0.52
J_w_	0.61	0.56	0.57	0.53	0.46	0.61
*Day 2*						
*Cut-off*	*176 U/L*	335 U/L*	126 mg/L*	*334 mg/L*	*0.204 µg/L*	1.280 µg/L*
Sensitivity	0.86	0.57	1.00	0.43	0.86	0.57
Specificity	0.42	0.93	0.61	0.99	0.52	0.94
LR +	1.49	8.34	2.53	32.6	1.78	8.80
LR-	0.34	0.46	0.00	0.58	0.28	0.46
DOR	4.43	18.1	2.53/0^a^	56.2	6.49	19.20
Youden	0.28	0.50	0.61	0.42	0.38	0.51
J_w_	0.43	0.62	0.74	0.60	0.49	0.63

*PPV*, positive predictive value; *NPV,* negative predictive value; *LR* + *,* positive likelihood ratio; *LR-,* negative likelihood ratio; *DOR*, diagnostic odds ratio; ^*^Same as the cutoff determined by the unweighted Youden’s index; ^a^Value cannot be calculated because the denominator is zero

**Fig. 3:**
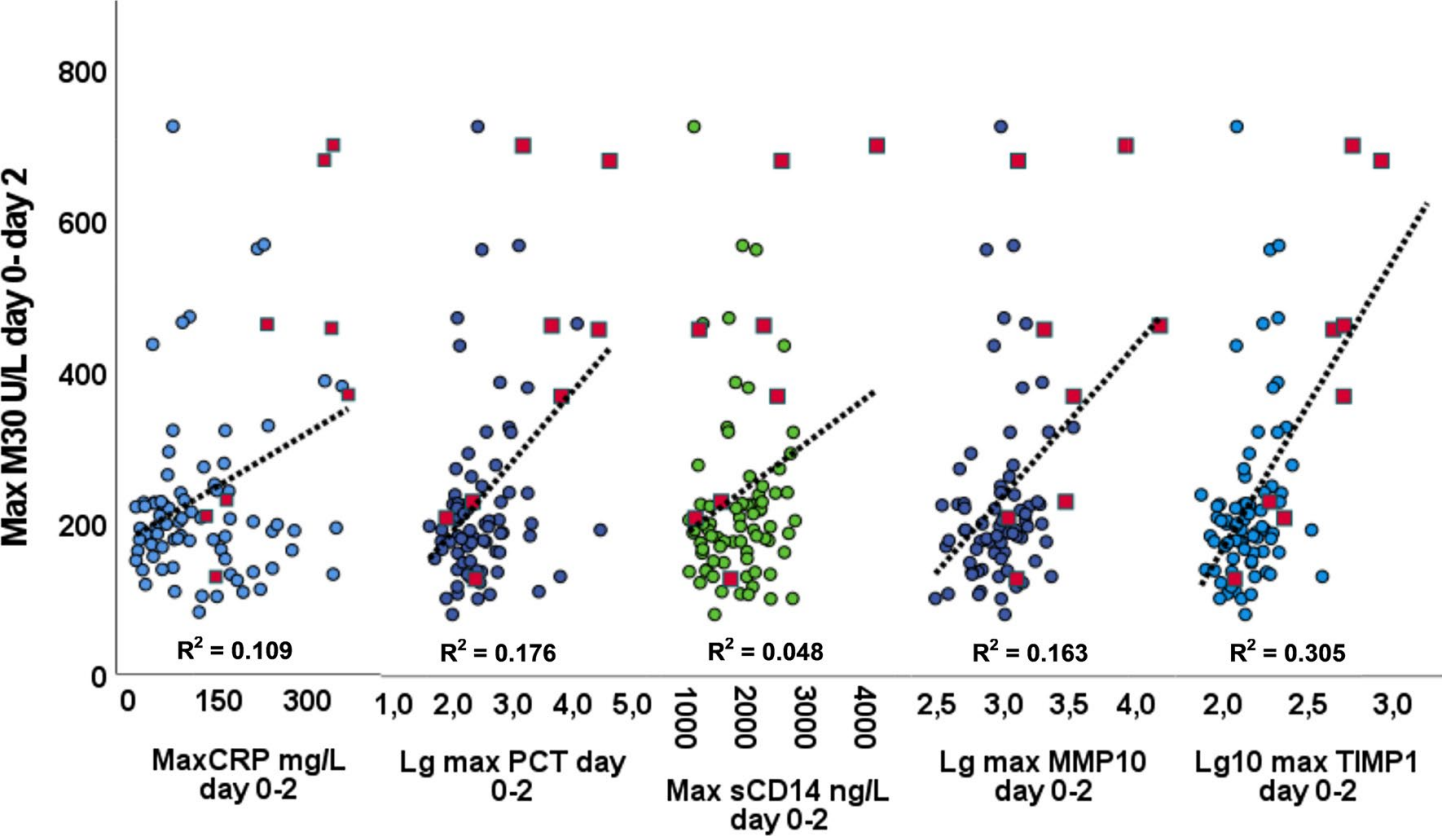
Relationship of cytokeratin-18 fragment M30 neoepitope with C-reactive protein (CRP), procalcitonin (PCT), soluble CD14, matrix metalloproteinase-10 (MMP10), and tissue inhibitor of metalloproteinase-1 (TIMP-1) in 86 hematological patients with severe sepsis (blue circles) or without severe sepsis (red squares). The coefficients of determination (R^2^) and regression lines for each variable are given

**Fig. 4:**
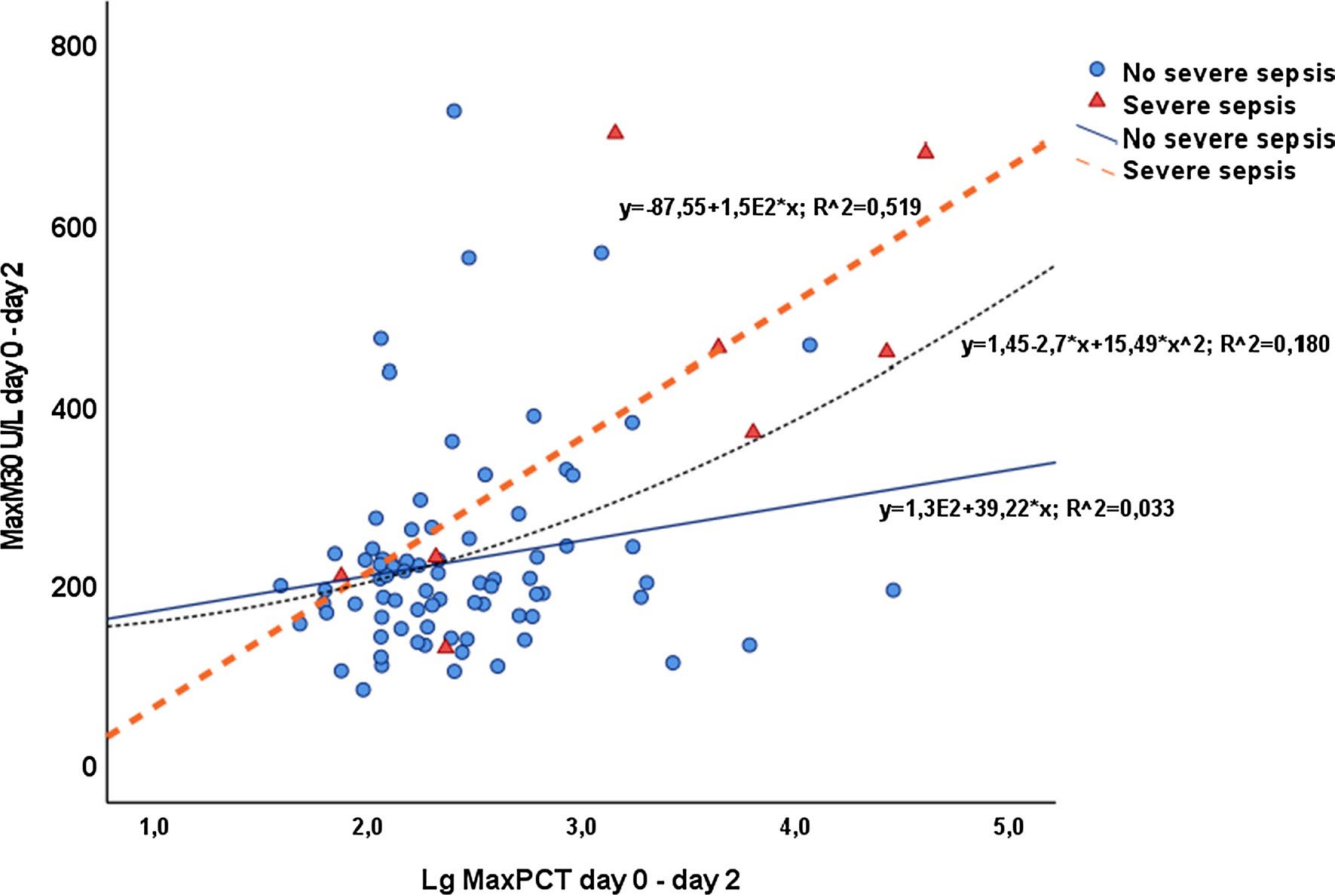
Relationship between maximum cytokeratin-18 d0-d2 and maximum procalcitonin d0-d2 in 86 hematological patients. Coefficient of determination (*R*^2^) was higher in patients developing severe sepsis (0.519) than in patients not developing severe sepsis (0.033). The linear-quadratic fit (*R*^2^ = 0.180) is given for all patients

To explore the role of CK-18 among other markers in FN, we investigated the correlation between CK-18 and CRP, PCT, tissue inhibitor of metalloproteinase-1 (TIMP-1), matrix metalloprotein-10 (MMP-10), and soluble CD14 subtype (sCD14), determined previously in the same patient population [32, 33]. The correlation of CK-18 fragment was highest with TIMP-1 (*r* = 0.431, *P* < 0.001), followed by that with MMP-10 (*r* = 0.351, *P* = 0.001), and PCT (*r* = 0.269, *P* = 0.012), while no significant correlation was observed with CRP or sCD14. Figure 3 shows the scatter plots for the relationships of cytokeratin-18 with CRP, PCT, MMP10, TIMP-1, and sCD14 in patients with severe sepsis and without severe sepsis. To further evaluate the association between CK-18 and PCT, the maximum d0-d2 CK-18 was plotted against the maximum d0-d2 logarithm-transformed PCT according to the development of severe sepsis (Fig. 4). The coefficient of determination (*R*^2^) was higher in patients developing severe sepsis (0.519) than in the rest of the patients (0.033).

## Discussion

This is the first study on cytokeratin-18 fragment M30 as an apoptosis-related prognostic marker in febrile neutropenia of hematological patients. The main finding of the study was that the level of M30 antibody detected fragments of CK-18 was higher on the first days of FN in patients who developed severe sepsis, septic shock or fatal outcome than in patients without these complications. On d1, which may be especially important from the clinician’s viewpoint, the performance of CK-18 was comparable with but not superior to that of CRP and PCT, and on d2 only CRP was predictive. On d0, in this patient population, none of these three markers was predictive for the development of severe sepsis. Blood culture positivity did not affect the level of CK-18.

Apoptosis has an important role in eliminating infected or damaged cells. The hallmark of apoptosis is the activation of caspase-3 protease which cleaves many different proteins including CK-18. Caspase-3 mediated apoptosis is associated with lymphocyte apoptosis in sepsis and thus possibly also with the impairment of immune response [34, 35]. Although apoptosis is considered as a non-inflammatory process of programmed cell death, apoptotic cells may be subject to secondary necrosis in the absence of effective clearance, thus inducing some inflammatory response [36], and possibly a rise in both apoptotic and inflammatory biomarker levels.

Even though the association between liver disease and elevated CK-18 is evident [4, 8, 9, 37] there is no evidence that in our study, the causal relationship of our main finding would be linked to hepatic failure. However, organ failure of severe sepsis might be a theoretical linkage between the severity of sepsis and elevated CK-18 but unlikely to be the only one.

We found slightly higher d0-d2 CK-18 levels in FN of NHL than in AML patients but the levels of CRP and PCT were similar in these two patient groups. Previously we observed higher levels of cell-free DNA (cfDNA) in patients with NHL than in patients with AML [38]. Also cfDNA is recognized to reflect apoptosis. Of note, CK-18 is not expressed in cells of lymphoid origin, and thus it could be used as a marker of treatment toxicity and gastrointestinal epithelial damage in lymphoma patients [39]. As previously observed, mucositis is found in a significant proportion of ASCT recipients at the time of FN [32]. In patients with lymphoma, Greystoke et al. observed an early difference in the CK-18 profiles between patients with and without clinical symptoms of toxicity, with the maximum level of CK-18 observed already on d3 while the clinical symptoms typically appeared on d7-d14 [39]. Thus, treatment toxicity-related epithelial damage is a plausible explanation for the difference in the CK-18 levels between AML and NHL patients observed in our study.

PCT is considered to have a better diagnostic capacity than CRP to predict complicated course of FN [40]. It also appears to be useful in distinguishing sepsis from non-infectious causes in FN [41]. In this study, the maximum d0-d2 CK-18 concentration correlated with PCT especially in patients with complicated FN. This finding is parallel to what was reported in a study with critically ill patients, where CK-18 level was associated with organ dysfunction, disease severity, and short-term mortality [22].

Meanwhile, the positive correlation between CK-18 and PCT was noted in our study of hematological patients with FN and in the study by Koch et al. including critically ill patients, no correlation between CK-18 and CRP was observed [22]. CRP is widely used to indicate infection, but it is a slow prognostic marker not peaking until 48–72 h after the onset of neutropenic fever [42]. As a future perspective, a combination of biomarkers with good diagnostic properties but without strong mutual correlation, thus reflecting different aspects of neutropenic infection, could be worth exploring.

There are some obvious limitations in this study. The main limitation of the study was the small number of FN patients with severe complications. In addition, we did not have the baseline CK-18 values from our patients before the start of intensive chemotherapy nor at the time of recovery from FN. The strengths of this study include the homogenous patient group consisting of neutropenic hematological patients with a recent history of intensive chemotherapy, early and well scheduled sampling, and prospective data collection for over 3 years. Of note, our FN study population had no previous liver diseases.

CK-18 is a potential early marker of severe complications in febrile neutropenia of hematological patients. Because of its role in apoptosis, it is of particular interest, complementing the spectrum of FN biomarkers. Further research is warranted to fully assess and understand the prognostic value of CK-18 in FN of hematological patients.

## Data Availability

The datasets generated during the current study are available from the corresponding author on reasonable request.
